# Presence of *Candida tropicalis* on *Staphylococcus epidermidis* Biofilms Facilitated Biofilm Production and *Candida* Dissemination: An Impact of Fungi on Bacterial Biofilms

**DOI:** 10.3389/fcimb.2021.763239

**Published:** 2021-10-22

**Authors:** Pornpimol Phuengmaung, Wimonrat Panpetch, Uthaibhorn Singkham-In, Tanittha Chatsuwan, Chintana Chirathaworn, Asada Leelahavanichkul

**Affiliations:** ^1^ Department of Microbiology, Faculty of Medicine, Chulalongkorn University, Bangkok, Thailand; ^2^ Antimicrobial Resistance and Stewardship Research Unit, Faculty of Medicine, Chulalongkorn University, Bangkok, Thailand; ^3^ Translational Research in Inflammation and Immunology Research Unit (TRIRU), Department of Microbiology, Faculty of Medicine, Chulalongkorn University, Bangkok, Thailand

**Keywords:** biofilms, *Staphylococcus epidermidis*, *Candida tropicalis*, infectious synergism, antibiofilm activity

## Abstract

While *Staphylococcus epidermidis* (SE) is a common cause of infections in implanted prostheses and other indwelling devices, partly due to the biofilm formation, *Candida tropicalis* (CT) is an emerging *Candida* spp. with a potent biofilm-producing property. Due to the possible coexistence between SE and CT infection in the same patient, characteristics of the polymicrobial biofilms from both organisms might be different from those of the biofilms of each organism. Then, the exploration on biofilms, from SE with or without CT, and an evaluation on l-cysteine (an antibiofilm against both bacteria and fungi) were performed. As such, *Candida* incubation in preformed SE biofilms (SE > CT) produced higher biofilms than the single- (SE or CT) or mixed-organism (SE + CT) biofilms as determined by crystal violet staining and fluorescent confocal images with z-stack thickness analysis. In parallel, SE > CT biofilms demonstrated higher expression of *icaB* and *icaC* than other groups at 20 and 24 h of incubation, suggesting an enhanced matrix polymerization and transportation, respectively. Although organism burdens (culture method) from single-microbial biofilms (SE or CT) were higher than multi-organism biofilms (SE + CT and SE > CT), macrophage cytokine responses (TNF-α and IL-6) against SE > CT biofilms were higher than those in other groups in parallel to the profound biofilms in SE > CT. Additionally, sepsis severity in mice with subcutaneously implanted SE > CT catheters was more severe than in other groups as indicated by mortality rate, fungemia, serum cytokines (TNF-α and IL-6), and kidney and liver injury. Although CT grows upon preformed SE-biofilm production, the biofilm structures interfered during CT morphogenesis leading to the frailty of biofilm structure and resulting in the prominent candidemia. However, l-cysteine incubation together with the organisms in catheters reduced biofilms, microbial burdens, macrophage responses, and sepsis severity. In conclusion, SE > CT biofilms prominently induced biofilm matrix, fungemia, macrophage responses, and sepsis severity, whereas the microbial burdens were lower than in the single-organism biofilms. All biofilms were attenuated by l-cysteine.

## Introduction

Biofilms are the irreversible attachment and growth of microorganisms on a surface using the bacterial extracellular polymers that promote attachment and matrix formation, resulting in an alteration in the microbial phenotype, especially in terms of growth rate, gene transcription, and antibiotic resistance (Otto, 2014). To survive in harsh environments, the microorganisms secrete several molecules and produce multilayers of high-abundance extracellular matrix (ECM) consisting of proteins, polysaccharides, and nucleic acids that are different among organisms and adhere onto the surface of some objects (non-living or biotic surfaces) (Otto, 2014). In staphylococcal biofilms, polysaccharide intercellular adhesin (PIA) or poly-β-1,6-linked N-acetylglucosamine (PNAG) is a major structural molecule of ECM (Lin et al., 2015). Because biofilm-residing bacteria can be resistant to the immune system and antibiotics, biofilm infections are usually chronic and difficult to treat (Otto, 2014). Although multidrug- or methicillin-resistant *Staphylococcus epidermidis* (MRSE) is an emerging healthcare problem (Lee et al., 2018), the biofilm-producing MRSE is even more difficult to treat compared with the regular MRSE (Neopane et al., 2018). With the increased use of indwelling medical devices, nosocomial infections caused by coagulase-negative staphylococci (CoNS), especially *S. epidermidis*, the most abundant microorganisms on human skin with biofilm-producing property, have become more common (Grice et al., 2009; Rogers et al., 2009; Sievert et al., 2013).

In parallel, catheter-related bloodstream infections (CRBSIs) with *Candida* spp. are also common in patients with chronic catheter insertion (Negri et al., 2012). Although *Candida albicans* are currently the most common causes of CRBSIs, reports on non-*albicans Candida* species (NACS), including *Candida tropicalis*, are increasing (Negri et al., 2012; Tan et al., 2018). Furthermore, *C. tropicalis* are common among patients with candidemia, specifically in Latin America and Asia (Zuza-Alves et al., 2017), and are identified as the most common *Candida* species among patients with candidemia in Thailand and even outperformed *C. albicans*: *C. tropicalis* (49.4%) *versus C. albicans* (28.8%) (Ngamchokwatthana et al., 2021). Interestingly, *C. tropicalis* is a virulent *Candida* species having a more potent biofilm-producing property than *C. albicans* with a wide range of virulence factors, including antifungal resistance and osmo-tolerance (an ability to survive in high salt concentrations) (Zuza-Alves et al., 2017).

Because of the high prevalence and the biofilm-producing property of *S. epidermidis* and *C. tropicalis*, the co-presentation of both organisms in biofilms is possible. Indeed, candidemia in patients at, before, or after staphylococcal septicemia and in patients with the simultaneous polymicrobial bloodstream infections from *Staphylococcus* spp. and *Candida* spp. is common (Bouza et al., 2013; Kim et al., 2013). Additionally, the combination of both organisms might produce some microbial benefits, as the diffusion of antimicrobial agents through polymicrobial biofilms of *Candida* and *S. epidermidis* is slower than the biofilms from each organism resulting an increased drug resistance (Adam et al., 2002; Hall and Mah, 2017; Bernard et al., 2020; Vila et al., 2021). Notably, the antibiotic diffusion through *C. tropicalis* biofilms is the slowest among all *Candida* species, indicating a possibility of the unique biofilm structures (AI-Fattani and Douglas, 2006). Although fungal biofilms are less common than bacterial biofilms, biofilms of both organisms correlate with the chronic infections and antibiotic resistance (Lynch and Robertson, 2008; Foreman et al., 2009). Moreover, the facilitation of biofilm production in *S. epidermidis* and *C. tropicalis* polymicrobial biofilms compared with the monomicrobial biofilms was demonstrated (Tan et al., 2018). However, the polymicrobial biofilms produced from the simultaneous attachment of both bacteria and fungi on catheter surface might be different from the biofilms from fungal attachment upon the preformed bacterial biofilms (fungemia after developed bacterial biofilms).

Accordingly, the formation of *C. albicans* biofilms on the preformed biofilms of *Pseudomonas aeruginosa* were more prominent than the biofilms from simultaneously mixed organisms that initially incubated together (Phuengmaung et al., 2020). Because of a few reports on polymicrobial *S. epidermidis* and *C. tropicalis* mixed biofilms and the possibility of the coexistence of both organisms (especially in South East Asia), the *in vitro* and *in vivo* biofilm models with the assessment microbial genes, macrophage responses against biofilms, and antibiofilm tests were conducted.

## Materials and Methods

### Animal

The animal study protocol (SST 012/2564) was approved by the Institutional Animal Care and Use Committee of the Faculty of Medicine, Chulalongkorn University, Bangkok, Thailand, following the National Institutes of Health (NIH), USA. Male C57BL/6 8-week-old mice were purchased from Nomura Siam International (Pathumwan, Bangkok, Thailand).

### Organism Preparation

Due to the limitation on biofilm production of laboratory strains, *S. epidermidis* and *C. tropicalis* were isolated from blood samples of patients from the King Chulalongkorn Memorial Hospital (Bangkok, Thailand) with the approval by the ethical institutional review board (610/2564), Faculty of Medicine, Chulalongkorn University according to the Declaration of Helsinki with written informed consent. Additionally, the laboratory standard for *S. epidermidis* (ATCC 12228; American Type Culture Collection, Manassas, VA, USA) was used in some experiments.

### *In Vitro* Biofilms and Antibiofilm

**Figure 1:**
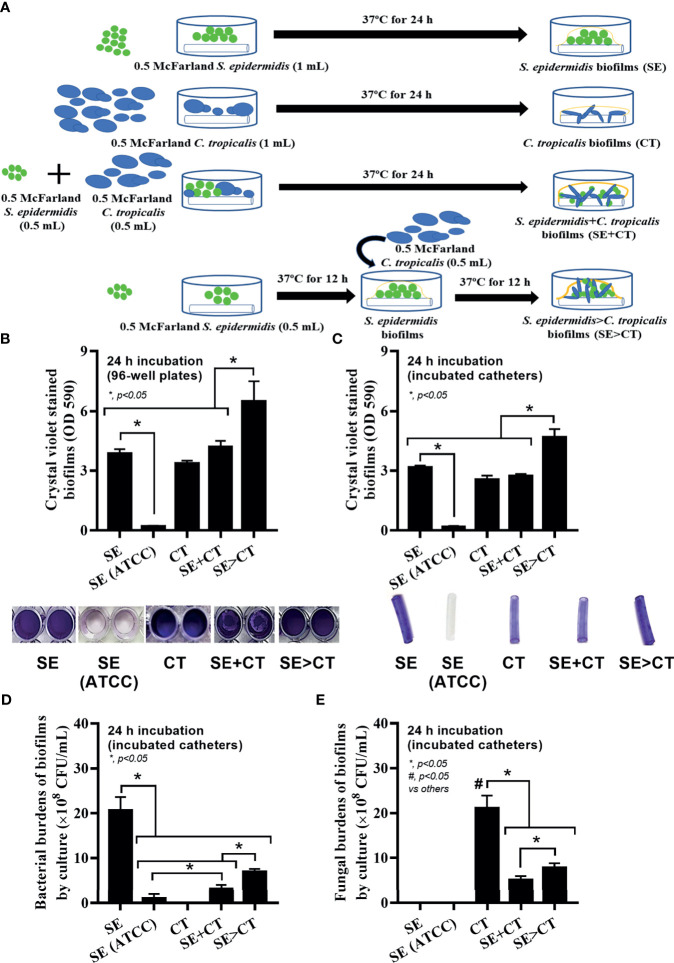
Schema of the *in vitro* experiments for the preparation of biofilms from *Staphylococcus epidermidis* (SE), *Candida tropicalis* (CT), SE simultaneously mixed with CT (SE + CT), and preformed SE biofilms following CT (SE > CT) **(A)** are demonstrated. Characteristics of biofilms from SE, either the clinically isolated strain (SE) or the standard strain [SE (ATCC)], CT, SE + CT, and SE > CT as determined by intensity of crystal violet color on 96-well plates and incubated catheters with the representative pictures **(B, C)**; bacterial and fungal burdens on catheter biofilms **(D, E)** are demonstrated (independent triplicate experiments were performed). The results were from three independent experiments in triplicate as the mean ± SEM; *p* < 0.05 was considered statistically significant. *p < 0.05 was considered statistically significant in the same group whereas #p < 0.05 was those in the other groups.

*S. epidermidis* (clinical isolation and ATCC strain) and *C. tropicalis* were grown in tryptic soy broth (TSB) and Sabouraud dextrose broth (SDB) (Oxoid, Cambridge, UK), respectively, at 37°C for 20 h to obtain the microorganisms in early stationary phase. Subsequently, the organisms were washed with pH 7.4 phosphate-buffered saline (PBS) and adjusted to the turbidity of 1 × 10^8^ CFU/mL (0.5 McFarland standard) in the culture media. Then, microbial suspension was transferred into flat-bottomed 96-well plates (200 μL/well) or 6-well plates (Sigma-Aldrich, St. Louis, MO, USA) containing 10-mm segments of polyurethane catheter (NIPRO, Ayutthaya, Thailand) (5 mL/well) and incubated at 37°C for 24 h before crystal violet staining as previously described (Phuengmaung et al., 2020). For the cover-glass biofilms, 5 mL of cell suspension was put in 6-well plates with an underlaid cover glass (22 × 22 mm; Thermo Fisher Scientific, Waltham, MA, USA) and incubated at 37°C before fluorescent color staining (Phuengmaung et al., 2020). There were three protocols of biofilm preparation, including i) monomicrobial biofilms using *S. epidermidis* or *C. tropicalis* at 0.5 McFarland standard 1 mL; ii) polymicrobial biofilms with the initial mixture of both organisms (SE + CT) using *S. epidermidis* and *C. tropicalis* and 0.5 mL of bacteria and fungi at 0.5 McFarland were combined into 1 mL; and iii) polymicrobial biofilms with *C. tropicalis* presentation on the previously formed SE biofilms (SE > CT) by incubating at half of reaction time using 0.5 mL of bacteria at 0.5 McFarland followed by 0.5 mL of fungi at 0.5 McFarland (**Figure 1A**). Both conditions of mixed-organism biofilms (SE + CT and SE > CT) were prepared with a half volume of each cell suspension at 1 × 10^8^ CFU/mL, whereas the single-organism condition was incubated with full volume of organism suspension at the same cell concentrations. Additionally, the potential ability of antibiofilms, l-cysteine (Sigma-Aldrich), was incubated together with the organisms using l-cysteine at the minimal biofilm inhibitory concentration (MBIC) of (50 mM) (**Supplementary Table 1**) per catheter before the evaluation of biofilms and microbial burdens.

### Evaluation of Minimal Biofilm Inhibitory Concentration and Minimal Inhibitory Concentration of l-Cysteine

MBIC and minimal inhibitory concentration (MIC) assay was performed by the broth microdilution method in 96-well flat-bottomed microplate format according to the Clinical and Laboratory Standards Institute (CLSI) guideline (Cruz et al., 2018; Clinical and Laboratory Standards Institute, 2020) with a minor modification. Briefly, microorganism suspension was diluted with TSB to obtain an inoculum of 1 × 10^6^ CFU/mL. Equal volumes of serial dilutions of l-cysteine ranging from 3.125 to 200 mM in Mueller Hinton Broth (MHB; Oxoid) and cell suspension were mixed and incubated at 37°C for 24 h. After incubation, MIC was determined by turbidity, and MBIC was subsequently defined by performing the crystal violet staining. For MBIC assay on catheter, equal volumes of microbial suspension and serial dilution of l-cysteine ranging from 3.125 to 200 mM in MHB was transferred into flat-bottomed 24-well plates containing 10-mm segments of polyurethane catheter and incubated at 37°C for 24 h before crystal violet staining.

### Quantification of Biofilms, Confocal Microscopy, and Biofilm Microbial Burdens

Biofilm production was determined by colorimetric crystal violet method, while the microbial burdens in the biofilms (bacteria and fungi) were determined by culture following a previous publication (Phuengmaung et al., 2020). In brief, the biofilms (on 96-well plates or in catheters) were stained with 0.1% crystal violet (Sigma-Aldrich), washed twice with deionized water, and dissolved with 30% acetic acid before determination of crystal violet intensity using absorbance at a wavelength of 590 nm of spectrophotometer (Thermo Fisher Scientific). For cover-glass biofilms, the biofilms were twice washed with PBS, fixed with 10% formalin for 10 min, and stained for i) bacterial DNA with 3.34 μM of SYTO9 (Invitrogen, Carlsbad, CA, USA); ii) ECM with 50 μg/mL of wheat germ agglutinin (WAG) conjugated with Alexa Fluor 488 (Invitrogen); and iii) fungi by 1 μg/mL of calcofluor white (CFW; Sigma-Aldrich) for 30 min in the dark before visualization using Zeiss LSM 800 Airyscan confocal laser scanning microscope (CLSM; Carl Zeiss, Jena, Germany). The fluorescent intensity was further evaluated by ZEN imaging software (Carl Zeiss). To determine organism burdens from biofilms, the biofilms were dissolved in normal saline (1 mL), thoroughly vortexed for 5 min, directly plated on tryptic soy agar (TSA) or Sabouraud dextrose agar (SDA) (Oxoid) in serial dilutions, and incubated at 37°C for 48 h before colony enumeration incubation. Of note, characteristics of colonies from bacteria and fungi were distinguishable on culture agar plates.

### *In Vitro* Experiments on THP-1-Derived Macrophages

Due to an importance of macrophages against inflammation and biofilms (Yamada et al., 2020), macrophage responses toward several groups of biofilms were determined. As such, the monocytic human cell line (THP-1) (ATCC TIB202) were prepared in Roswell Park Memorial Institute (RPMI) 1640 medium supplemented with 10% heated-inactivated fetal bovine serum (FBS; Gibco BRL/Life Technologies, Ltd., Paisley, Scotland, UK) at 37°C in a humidified CO_2_ incubator. Phorbol myristate acetate (PMA; Sigma-Aldrich) at a final concentration of 100 nM was added to differentiate it into macrophages. The cells were washed with Hanks’ balanced salt solution (HBSS; Gibco BRL) and rested in fresh PMA-free FBS medium at 37°C overnight. The trypan blue exclusion method was used to determine the cell viability by counting stained (dead cells) *versus* unstained cells (live cells) on a hemocytometer. The preparation with cell viability >95% was used for the further experiments. With an effector-to-target ratio (E:T) of 10:1, macrophages at 1 × 10^6^ cells/mL with biofilm (forming by 1 × 10^5^ organisms in each biofilm condition) that was thoroughly removed from 96-well plates by scraping was incubated in 37°C incubator with 5% CO_2_ for 4 h. Then, supernatant cytokines (TNF-α, IL-6, and IL-10) were determined by ELISA, and several Toll-like receptors (*TLR*s) were evaluated by quantitative reverse transcription–PCR (qRT-PCR). The list of primers for PCR is presented in **Supplementary Table S2**.

### Quantitative Real-Time Polymerase Chain Reaction

Total RNA was extracted from organisms and THP-1 cells using TRIzol reagent (Ambion, CA, USA) with RNase-free DNase I (Thermo Fisher Scientific). The RNA purity and quantity were determined by the absorbance at 260 and 280 nm. Purified RNA was converted by reverse transcriptase (RevertAid First Stand cDNA) and conducted qRT-PCR on Applied Biosystems QuantStudio 6 Flex Real-Time PCR System (Applied Biosystems, Warrington, UK) with SYBR^®^ Green PCR master mix. The housekeeping genes, *16S rRNA* and *β-actin*, were used to normalize the transcriptional levels of target genes from *S. epidermidis* and macrophages, respectively, with the comparative cycle threshold against the expression. The list of primers for PCR is presented in **Supplementary Table S2**.

### *In Vivo* Experiments

Subcutaneous implantation of catheters with biofilms in different conditions was performed in mice following a previous publication (Phuengmaung et al., 2020). Briefly, 10-mm catheters (NIPRO) were cultured with the early stationary phase of organisms (*S. epidermidis* alone, *C. tropicalis* alone, and the mixed organisms of *S. epidermidis* plus *C. tropicalis*) at 37°C for 4 h prior to subcutaneous implantation in both sides of mouse flank area under isoflurane anesthesia. For the *S. epidermidis* > *C. tropicalis* condition, *S. epidermidis* was incubated with catheters at 37°C for 2 h before the administration of *C. tropicalis* and further incubated for 2 h prior to catheter insertion into mice. In the antibiofilm-treated groups, l-cysteine (Sigma-Aldrich) at 50 mM per catheter was incubated together with the organisms. Then, mice were sacrificed at 96 h by cardiac puncture under isoflurane anesthesia with sample collection and observed for 120-h survival analysis. Biofilms and microbial burdens in catheters were determined by crystal violet and culture method as described above. For bacteremia and fungemia, 100 μL of blood was spread onto TSA and SDA (Oxoid) at 37°C for 24-h incubation before colony enumeration. Serum cytokines were evaluated by ELISA (Invitrogen), while renal and liver injury were determined by serum creatinine and alanine transaminase using QuantiChrom creatinine assay (DICT-500) (BioAssay, Hayward, CA, USA) and EnzyChrom Alanine Transaminase assay (EALT-100) (BioAssay), respectively.

### Statistical Analysis

Mean ± standard error (SE) was used for data presentation. The differences between groups were examined for statistical significance by one-way analysis of variance (ANOVA) followed by Tukey’s analysis or Student’s t-test for multiple groups comparison or two groups comparison, respectively. Survival analysis and the time-point experiments were analyzed by log-rank test and the repeated-measures ANOVA, respectively. All statistical analyses were performed with SPSS 11.5 software (SPSS, IL, USA) and GraphPad Prism version 7.0 software (La Jolla, CA, USA). A *p*-value of <0.05 was considered statistically significant.

### Data Availability Statement

The raw data supporting the conclusions of this article will be made available by the authors, without undue reservation, to any qualified researcher.

## Results

### The Presence of *Candida tropicalis* on Preformed Biofilms of *Staphylococcus epidermidis* Facilitated Biofilm Production

**Figure 2:**
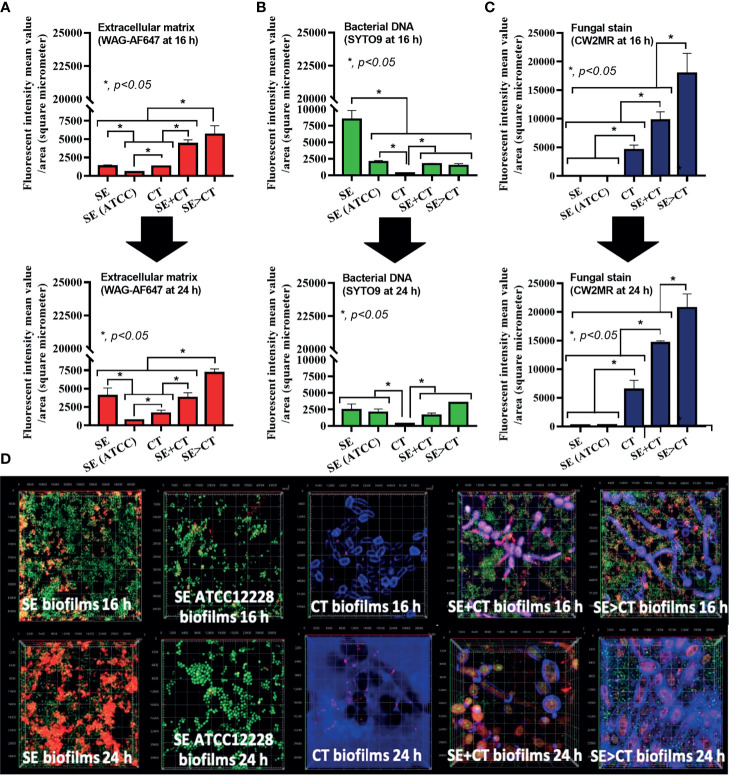
Characteristics of biofilms from *Staphylococcus epidermidis* (SE) using the clinically isolated strain (SE) or the standard strain [SE (ATCC)], *Candida tropicalis* (CT), SE simultaneously mixed with CT (SE + CT), and preformed SE biofilms following by CT (SE > CT) as determined by intensity of fluorescent stains from cover-glass biofilms at 16 h (top) and 24 h (bottom) using wheat germ agglutinin (WAG)-AF647 (red color fluorescence) for extracellular matrix **(A)**, SYTO9 (green color fluorescence) for bacterial nucleic acid **(B)**, and calcofluor white (CW2MR) (blue color fluorescence) for fungal cell wall **(C)**, with the representative fluorescent images **(D)** demonstrated (independent triplicate experiments were performed). The results were from three independent experiments in triplicate as the mean ± SEM; *p* < 0.05 was considered statistically significant.

**Figure 3:**
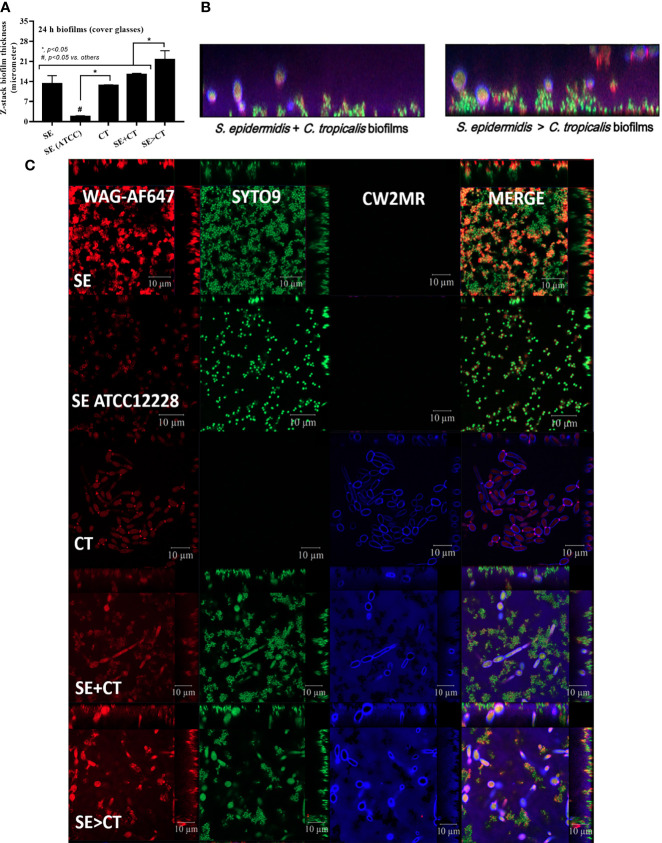
Biofilm thickness as evaluated by z-stack analysis of fluorescent images from 24-h biofilms on cover glasses of *Staphylococcus epidermidis* (SE) using the clinically isolated strain (SE) or the standard strain [SE (ATCC)], *Candida tropicalis* (CT), SE simultaneously mixed with CT (SE + CT), and preformed SE biofilms followed by CT (SE > CT) as indicated by the fluorescent score using wheat germ agglutinin (WAG)-AF647 (red color fluorescence) for extracellular matrix, SYTO9 (green color fluorescence) for bacterial nucleic acid, and calcofluor white (CW2MR) (blue color fluorescence) for fungal cell wall, with the representatives fluorescent images **(A–C)** demonstrated (independent triplicate experiments were performed). The results were from three independent experiments in triplicate as the mean ± SEM; *p* < 0.05 was considered statistically significant.

The procedures for single-microbial biofilms and multi-organism biofilms are shown in **Figure 1A**. The standard strain of *S. epidermidis*, SE (ATCC 12228), could not produce biofilms in 96-well plates, catheters, and fluorescent-stained cover glasses, which resulted in the microbial burdens that were less than those of the clinically isolated *S. epidermidis* (SE) (**Figures 1B–E**, **2A–D**, and **3A, C**). Although some organisms in biofilms could not be cultured (Moser et al., 2021), the microbial burdens on biofilms were roughly determined by culture (**Figures 1D, E**), and the burdens on biofilm surface were evaluated by confocal images (**Figures 1A–D**). With crystal violet staining at 24 h of incubation, the intensity of monomicrobial biofilms (SE or CT) and biofilms from initially mixed organisms (SE + CT) was not different (**Figures 1B, C**), whereas the biofilms forming *Candida* on preformed bacterial biofilms (SE > CT) were more prominent than SE + CT biofilms (**Figures 1B, C**). In contrast, microbial burdens of the single-organism biofilms (SE and CT) were higher than those of the mixed-organism biofilms (SE + CT and SE > CT) (**Figures 1D, E**), possibly due to the difference of the initial amount of bacteria in each protocol (**Figure 1A**). These data support an enhanced ECM production despite the limited numbers of organisms during the biofilm production. Likewise, confocal microscopic images of cover-glass biofilms indicated more prominent ECM in multi-microbial biofilms (SE + CT and SE > CT), with the highest ECM in the SE > CT group, when compared with monomicrobial biofilms (SE and CT) at 16 and 24 h of incubation using WAG staining (**Figure 2A**). For bacterial DNA staining using SYTO9 color, bacterial DNA on SE biofilm surface was higher than that of other groups at 16 h of incubation and has no difference to that of other SE-containing biofilms at 24 h post-incubation (**Figure 2B**). Bacteria on the surface of SE + CT biofilms were similar to those of SE > CT biofilms in both time-points (**Figure 2B**), whereas the bacterial burdens by culture in SE > CT biofilms were higher than in the SE + CT group (**Figure 1D**). In parallel, fungal staining by CFW (CW2MR) indicated the highest stained fungi on the surface of SE > CT biofilms when compared with SE + CT or CT alone biofilms, and the biofilms from CT alone demonstrated the lowest fungi on biofilm surface among CT-containing biofilms (**Figure 2C**), suggesting a good attachment between fungi and the surface material. Conversely, fungal burdens by culture in CT biofilms were more prominent than in other fungal-containing biofilms (**Figure 1E**), implying the different aspects between culture (total microbial burdens) and confocal image analysis (microbial burdens on each area of biofilm surface). Meanwhile, the thickness of biofilms as determined by z-stack fluorescent image analysis demonstrated the most thickness in SE > CT biofilms, while the thickness of other groups, except for the standard stain SE, was similar (**Figures 3A–C**). Furthermore, the lateral view of z-stack images also supported more prominent thickness of SE > CT biofilms than SE + CT biofilms (**Figure 3B**).

### *Candida tropicalis* Facilitated *Staphylococcus epidermidis* Biofilms Possibly Through the Downregulation and Upregulation of Genes for Biofilm Suppression and Adhesive Proteins, Respectively

**Figure 4:**
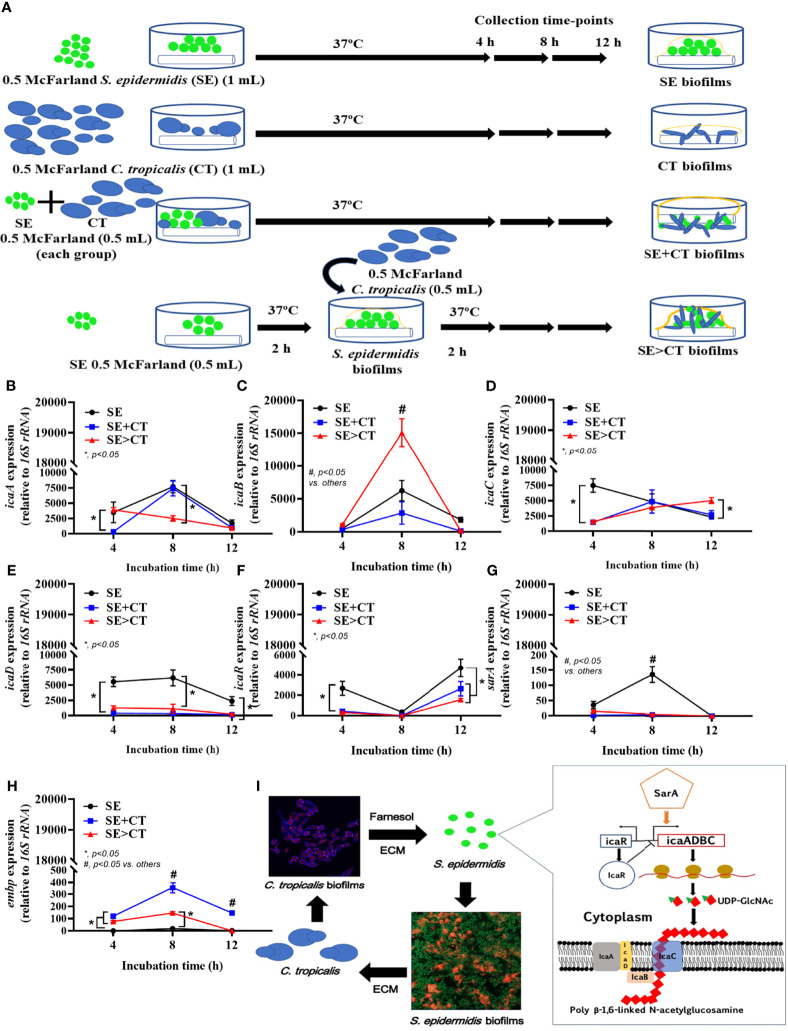
The representative pictures of biofilm thickness by the lateral view of z-stack fluorescent stained pictures from clinically isolated *Staphylococcus epidermidis* (SE) and *Candida tropicalis* (CT) mixing (SE + CT) (left side) and SE before CT (SE > CT) (right side), indicating bacterial nucleic acid (SYTO9; green color fluorescence), extracellular matrix (wheat germ agglutinin (WAG)-AF647; red color fluorescence), and fungal cell wall (CW2MR; blue-purple color fluorescence) **(A)** are shown. Expression of SE biofilm-associated genes from cover-glass biofilms of clinically isolated SE (SE), *Candida tropicalis* (CT), SE simultaneously mixed with CT (SE + CT), and preformed SE biofilms following by CT (SE > CT) in different time-points **(B–G)** are demonstrated (independent triplicate experiments were performed). Diagram of the possible linkage between CT and SE biofilm synthesis indicates that *Candida*-producing farnesol, a quorum-sensing molecule blocking the yeast-to-filament conversion in fungal biofilms (Carolus et al., 2019), stimulates SE biofilm synthesis through the upregulated *sarA* and downregulated *icaR* (as demonstrated in the pathway) (Brescó et al., 2017) **(H)**. Notably, *icaA* and *icaD* are responsible for UDP-Glc-NAc [uridine diphosphate *N*-acetylglucosamine, a single unit of the polysaccharide intercellular adhesin (PIA) in extracellular matrix (ECM)], while *icaB* and *icaC* are used for polymerization and transportation of ECM, respectively (Arciola et al., 2015) and the possible association with *Candida* **(I)**. Meanwhile, *embp* expression increases bacterial adhesive protein of ECM. The results were from three independent experiments in triplicate as the mean ± SEM; *p* < 0.05 was considered statistically significant.

Due to the previous reports on facilitation of bacterial biofilms by *Candida* spp. (Rodrigues et al., 2019; Phuengmaung et al., 2020; Luo et al., 2021), expression of several genes of *S. epidermidis* biofilms production was explored (**Figures 4A–I**). The synthesis pathway for PIA, a polymer of uridine diphosphate N-acetylglucosamine (UDP-Glc-NAc) of *S. epidermidis* ECM, and the possible association with *Candida*, is demonstrated in **Figure 4I**. The important PIA-associated genes are i) *icaADBC*-encoded proteins; ii) *Staphylococcus* accessory regulators (*sar*), especially *sarA*; iii) the negative regulator from intercellular adhesin locus regulator (*icaR*) (Tormo et al., 2005; Brescó et al., 2017); and iv) the ECM binding proteins encoded by *embp* for surface attachment and fibronectin bridging (Linnes et al., 2013). At 4-h incubation, staphylococcal *icaA* and *icaD* genes (UDP-Glc-NAc synthesis), *icaC* (polymerization process), and *icaR* (ECM suppressor), except for *icaB* (deacetylation), *sarA* (ECM enhancer), and *embp* (ECM adhesive proteins), were upregulated in SE biofilms when compared with the mixed biofilms (SE + CT and SE > CT) (**Figures 4B–I**). Subsequently, the mixed biofilms (SE + CT and SE > CT) upregulated *icaADBC* (except for *icaD*), *icaR*, and *embp*, but not *sarA*, at 8- or 12-h incubation when compared with SE biofilms (**Figures 4B–I**), indicating that the addition of CT may relate to the enhanced ECM synthesis (*icaADBC*) with the complete biofilm structure (*embp*) rather than the ECM enhancement through *sarA*. There was higher expression of *icaB* (8 h) and *icaC* (12 h) in SE > CT biofilms than in other groups (**Figures 4B–I**), which might be associated with the prominent SE > CT biofilms (**Figures 1B, C**). These data suggest that the prominent ECM in SE > CT over SE + CT biofilms was possibly due to the deacetylation (*icaB*) and matrix polymerization (*icaC*), in contrast to the synthesis processes. Despite the profound *embp* expression in SE + CT biofilms (8–12 h) compared with other groups (**Figure 4H**), SE + CT biofilms did not demonstrate the prominent biofilms (**Figures 1B, C**), which supported the requirement of several genes for biofilm synthesis. Nevertheless, these data imply a facilitation of SE biofilms by *C. tropicalis* in some processes of matrix production and fibronectin bridging of the biofilms.

### Profound Macrophage Responses Against Multi-Organism Biofilms From *Candida tropicalis* on the Preformed *Staphylococcus epidermidis* Biofilms But Attenuated by l-Cysteine

**Figure 5:**
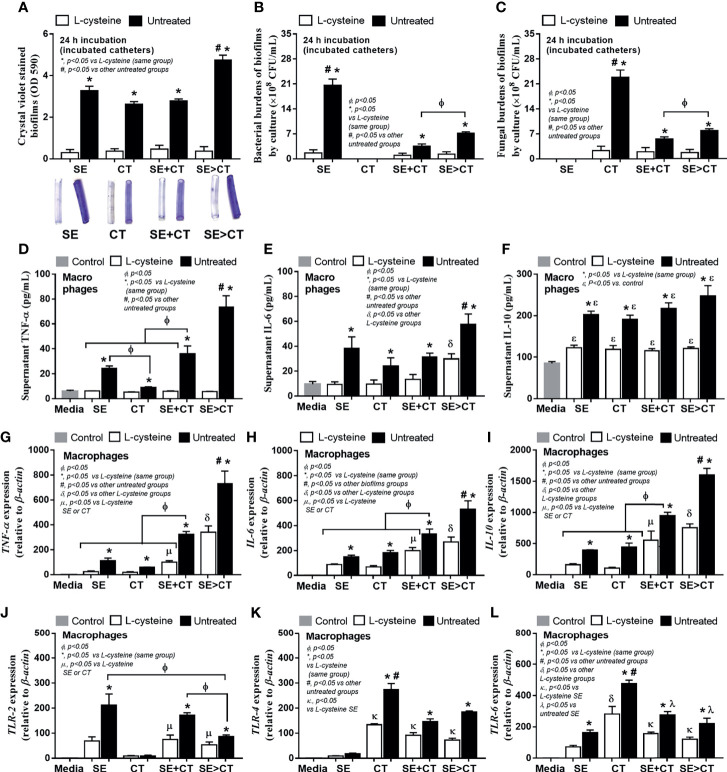
Characteristics of catheter biofilms from clinically isolated *Staphylococcus epidermidis* (SE), *Candida tropicalis* (CT), SE simultaneously mixed with CT (SE + CT), and preformed SE biofilms following by CT (SE > CT) with l-cysteine (50 mM) as determined by crystal violet intensity **(A)**; and microbial burdens (bacteria and fungi) by culture from the biofilms **(B, C)** are demonstrated (independent triplicate experiments were performed). The responses of macrophages against biofilms from different groups as determined by cytokines (TNF-α, IL-6, and IL-10) in supernatant **(D–F)** with expressed genes for cytokines (*TNF-α*, *IL-6*, and *IL-10*) **(D–I)** and inflammatory receptors (*TLR-2*, *TLR-4*, and *TLR-6*) **(J–L)** are demonstrated (independent triplicate experiments were performed). Notably, the control groups (Control) were macrophages incubated with only culture media, while all gene expressions were calculated relative to the control group.

Because of i) the antibiofilm property of l-cysteine (Dinicola et al., 2014), ii) a well-known influence of macrophages on biofilm-associated molecular patterns (Thurlow et al., 2011; Moser et al., 2021), and iii) the enhanced macrophage responses against mixed microbial molecules from bacteria and fungi (Panpetch et al., 2017; Panpetch et al., 2019; Phuengmaung et al., 2020), l-cysteine and macrophage responses were tested. As such, l-cysteine similarly attenuated biofilms (crystal violet staining) and reduced microbial burdens (culture method) in all groups (**Figures 5A–C**) possibly due to the loss of microbial attachment to catheters from l-cysteine effect. Regarding macrophage responses, SE > CT biofilms induced the most prominent inflammatory responses among all groups as determined by supernatant TNF-α and IL-6 (but not IL-10) and gene expression of TNF-α, IL-6 and IL-10 (**Figures 5D–I**). Additionally, macrophage inflammatory responses were more profound in mixed-organism biofilms (SE + CT and SE > CT), when compared with single-organism biofilms (**Figures 5D–I**). The macrophage responses against SE monomicrobial biofilms were similar to those of CT, except for the highest secretion of TNF-α after activation by SE biofilms (**Figures 5D–I**). Notably, the increased prominent macrophage responses toward biofilms of mixed organisms are more comparable with single-organism biofilms (**Figures 5D–I**), despite a lower microbial abundance of multi-organism biofilms (**Figures 5B, C**), implying the possible profound responses against the molecules from biofilm structures over the organismal molecules. However, the expression of *TLR*s on macrophages was not dominant on mixed-organism biofilms, as *TLR-4* and *TLR-6* were the most dominant in responses against CT monomicrobial biofilms and *TLR-2* was high in both SE and SE + CT biofilms (**Figures 5J–L**), suggesting a possible influence of other inflammatory pathways against biofilm-associated molecular patterns (Moser et al., 2021). Nevertheless, l-cysteine attenuated the responses of macrophages in all parameters (**Figures 5D–L**), perhaps because of the reduction in either microbial burdens or biofilm-associated molecules (**Figures 5B, C**).

### Severe Sepsis in Mice With Subcutaneous Implantation of Biofilm Catheters Using *Candida tropicalis* on Preformed *Staphylococcus epidermidis* Biofilms and the Attenuation by l-Cysteine

**Figure 6:**
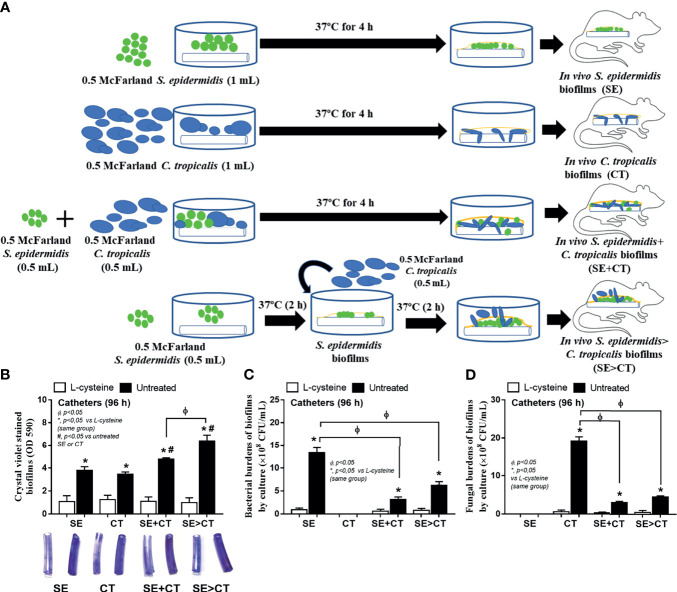
Schema of the *in vivo* experiments for the subcutaneous implantation of catheters with biofilms from *Staphylococcus epidermidis* (SE), *Candida tropicalis* (CT), SE simultaneously mixed with CT (SE + CT), and preformed SE biofilms following CT (SE > CT) **(A)** demonstrated. Characteristics of mice with subcutaneously implanted catheters with l-cysteine or untreated groups as determined by biofilms in catheters (intensity of crystal violet color) **(B)** and microbial burdens by culture (bacteria and fungi) in biofilms **(C, D)** (n = 7–9/group) are demonstrated.

**Figure 7:**
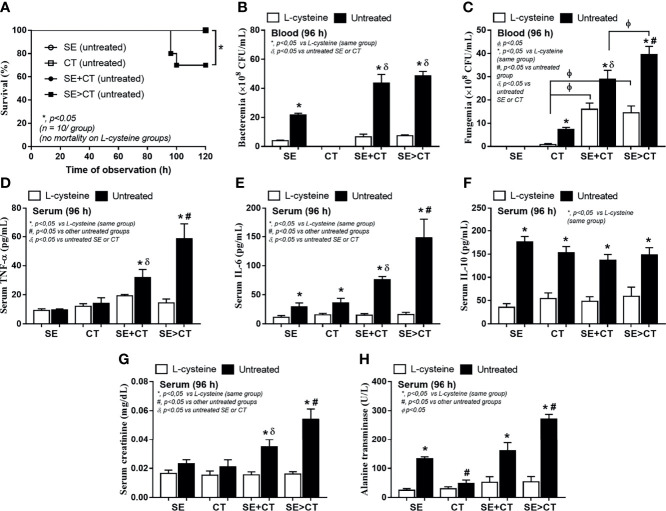
Characteristics of mice with subcutaneously implanted biofilm catheters from *Staphylococcus epidermidis* (SE), *Candida tropicalis* (CT), SE simultaneously mixed with CT (SE + CT), and preformed SE biofilms following CT (SE > CT) as determined by survival analysis **(A)**, bacteremia and fungemia **(B, C)**, serum cytokines (TNF-α, IL-6, and IL-10) **(D–F)**, kidney injury (serum creatinine) **(G)**, and liver damage (alanine transaminase) **(H)** are demonstrated (n = 7–9/group for **B–H**). *p < 0.05 was considered statistically significant in the same group whereas ^#^p < 0.05 was those in the other groups.

To initially evaluate an *in vivo* impact of the biofilms from different groups, catheters with biofilms from different protocols were subcutaneously implanted in mice (**Figure 6A**). Because of an early expression of biofilm-associated genes (within 4–6 h post-incubation) (Kot et al., 2018; Phuengmaung et al., 2020), SE was incubated only 2 h before adding CT in the SE > CT group (**Figure 6A**) that was different from the prior experiments (**Figure 1A**). After 96 h of subcutaneous implantation, SE > CT biofilms demonstrated the most prominent intensity than other groups with the lower organismal burdens than the single-organism biofilms (**Figures 6B–D**). Microbial burdens in SE + CT and SE > CT biofilms in mice were not different (**Figures 6C, D**), despite that the microbial burdens of SE > CT were higher than in SE + CT biofilms in the *in vitro*-incubated catheters at 24 h (**Figures 5B, C**), possibly due to the less limited source of microbial nutrients in mice than the *in vitro* catheter biofilms (Phuengmaung et al., 2020). However, l-cysteine attenuated biofilms and microbial burdens in all groups of mice (**Figures 6B–D**) were similar to the *in vitro* catheter biofilms using the incubators (**Figures 5A–C**). Only mice with SE > CT biofilm catheters developed severe sepsis as indicated by 30% mortality rate in this group, while there was no mortality in other biofilm groups (**Figure 7A**), possibly due to the most prominence (among all groups) in the level of fungemia (but not bacteremia), systemic cytokines (TNF-α and IL-6 but not IL-10), renal injury (serum creatinine), and liver damage (alanine transaminase) (**Figures 7A–H**). Additionally, mice with mixed-biofilm catheters (SE + CT and SE > CT) demonstrated the most severe infection when compared with monomicrobial biofilm catheters as indicated by these parameters, except for serum IL-10 (**Figures 7A–H**). Of note, fungemia in mice with SE > CT biofilm catheters was more prominent than in the SE + CT groups (**Figures 7B, C**), which might be responsible for the higher mortality in the SE > CT group (**Figure 7A**). These data supported the sepsis severity and the high mortality rate of fungemia-induced sepsis when compared with bacterial sepsis (Moyes and Naglik, 2011; Delaloye and Calandra, 2014). Nevertheless, biofilm prevention with l-cysteine attenuated all sepsis parameters in all groups of mice with biofilm catheters (**Figures 7A–H**), except for the incomplete reduction of fungemia in mice with SE + CT and SE > CT biofilm catheters (**Figure 7C**). These data support the enhanced microbial eradication by host immunity after the blockage of biofilm formation (Gebreyohannes et al., 2019) and a possible effect of l-cysteine for the biofilm prevention (Blasi et al., 2016; Kregiel et al., 2019).

## Discussion

### *Candida tropicalis* Presentation on Preformed *Staphylococcus epidermidis* Biofilms Enhanced Biofilm Production Through the Interference on Staphylococcal *icaADBC* Expression

The possible coexistence between *S. epidermidis* (SE) (Brescó et al., 2017) and *C. tropicalis* (CT) (Negri et al., 2012), the enhanced biofilms of *Staphylococcus* spp. and *Candida* spp. (El-Azizi et al., 2004; Dinicola et al., 2014), the increased morbidity of mixed-biofilm catheter infection (Kong et al., 2015; Nash et al., 2016), and the mixed biofilms were studied. Here, biofilm production from mixed organism (SE + CT) was not different from the biofilm SE or CT alone, in contrast to the numerous biofilms from CT upon preformed SE biofilms (SE > CT), suggesting the possible different structures of SE > CT biofilm. Polysaccharides type I and II, referred to as PIA and PNAG, respectively, are the main structure of SE biofilms (Mack et al., 1996), which are synthesized through *icaADBC* genes (Heilmann et al., 1996; Mack et al., 2007; Arciola et al., 2015; Harris et al., 2016) and regulated (aggregation and maturation) by various genes, including staphylococcal accessory homologous A (*sarA*; inducible by salt water and harsh environments) (Christner et al., 2012; Carolus et al., 2019) (**Figure 4I**). As such, *sarA* binds to *icaA* promoter on *icaADBC* leads to *icaA* and *icaD* co-expression for exopolysaccharide PIA synthesis (Gerke et al., 1998); then *icaC* induces enzymes for PIA transfer to bacteria surface, *icaB* products operate deacetylation of the polymers (Vuong et al., 2004), and ECM-binding protein encoded gene (*embp*) (also controlled by *sarA*) induces surface attachment (Hussain et al., 2001).

On the other hand, the structures of *Candida* biofilms also consist of the similar polysaccharides, making it possible for the physical biofilm interaction between these organisms (Carolus et al., 2019). Additionally, Farnesol, a *Candida* molecule for the inhibition of the mold to yeast transformation, promotes staphylococcal ECM production in *Candida*–*Staphylococcus* polymicrobial biofilms (Krause et al., 2015). Meanwhile, staphylococci also enhance *Candida* biofilm formation (Vila et al., 2019) and predominantly attach to the fungal hyphae in the mixed-organism biofilm architecture (Peters et al., 2012). Here, *Candida* developed from yeast to hyphal form in both SE + CT and SE > CT biofilms as visualized by confocal images in both groups. Perhaps, preformed SE biofilms in the SE > CT group enhanced fungal biofilm formation, and the hyphal invasion in the SE > CT group promotes the production of SE ECM (Sudbery et al., 2004). On the other hand, SE + CT biofilms had neither the stimulation by preform SE biofilms nor biofilm bleaching by fungal hyphae, which resulted in less biofilm production than SE > CT biofilms. While *icaADBC* (PIA-promoting proteins) and *icaR* (PIA inhibitor) genes are on the same operon, *icaADBC* is transcribed as a polycistronic mRNA (an mRNA that encodes two or more proteins). The products of *icaA* alone could promote PIA synthesis, and *icaA* co-expressed with *icaD* synergistically produces PIA oligomers (Wojtyczka et al., 2014). Additionally, the deacetylation (*icaB*) and matrix polymerization (*icaC*) of PIA are also required for ECM production, as the loss of *icaB* reduces PIA cationic property that worsens the ability to attach to the bacterial cell surface (Vuong et al., 2004). Despite a polycistronic mRNA of *icaADBC* with the same starting operon, these genes seem to be presented in a different period of the incubation (from our results), and the discordance on the expression of these genes is possible, as some strains of bacteria express only *icaA* and *icaD* (Wojtyczka et al., 2014; Ghasemian et al., 2016). On the other hand, *icaR* gene (PIA inhibitor), which locates adjacent to the *icaADBC* operon in the opposite direction, is a member of the *tetR* family of transcriptional regulators that are activated by several stimulators (Conlon et al., 2002). The upregulation of *icaB* and *icaC* in *icaADBC* complex in SE > CT was more prominent than in the SE + CT group. Although *sarA* and *icaD* (PIA synthesis) in SE biofilms were higher in the SE > CT group, *icaB* (PIA polymerization) and *icaC* (PIA transportation) in SE biofilms were lower than in the SE > CT group. Thus, the less biofilm intensity in SE alone than in the SE > CT group might be due to the enhanced PIA polymerization and transportation that strengthen the biofilm structures. Likewise, the lower PIA inhibitory products of *icaR* in SE + CT or SE > CT compared with SE alone might be also important. Hence, our preliminary results implied that *Candida* presentation upon SE biofilms enhanced the biofilm formation, partly, through the facilitation on biofilm production, in particular PIA polymerization and transportation, with the reduced PIA repressors. Notably, there was a difference between the duration of SE pre-incubation before adding CT in the SE > CT group in gene expression (2 h) (**Figure 6A**) *versus* imaging experiments (12 h) (**Figure 1A**) because the expression of biofilm-associated genes was very early (within 4–6 h of microbial attachment) (Kot et al., 2018; Phuengmaung et al., 2020). Then, the maximal incubation time of SE incubation before adding CT was at 2 h (half of the incubation time for other groups). However, the 2-h SE pre-incubation was enough for an initial SE biofilm formation as indicated by crystal violet staining (data not shown). Despite the absence of a clear conclusion on the gene expression experiments, our findings provided proof of concept for an impact of *Candida* on SE-preformed biofilms. More studies in this topic are warrant.

### *Candida tropicalis* Presentation Upon Preformed *Staphylococcus epidermidis* Biofilms Induced the More Prominent Macrophage Responses and More Severe Sepsis Than Other Groups

Macrophages are important for responses against several foreign molecules (Akira and Hemmi, 2003), including biofilms, as TLR-2 and TLR-6 heterodimers recognize PIA from SE biofilms (Hanke and Kielian, 2012; Fournier, 2013), whereas *Candida* activates TLR-2 and TLR-4 (Yuan and Wilhelmus, 2010). Here, the SE > CT group produced the highest biofilm intensity among all groups and induced the highest macrophage pro-inflammatory cytokines (TNF-α and IL-6), despite the lower microbial abundance than monomicrobial biofilms. These data imply the potent inflammatory activation from biofilm-associated molecular patterns (Moser et al., 2021) involved more than the molecules from planktonic form of bacteria. Indeed, the immune responses against biofilm exopolysaccharides, especially the pathogenic bacterial biofilms (Littman and Pamer, 2011), are stronger than the planktonic polysaccharide responses (Rybtke et al., 2020; Moser et al., 2021), despite the lower polysaccharide production in pathogens than normal flora (Comstock and Kasper, 2006). Certainly, the purified PIA from SE biofilms partly induces TLR-2 (Stevens et al., 2009; Fredheim et al., 2011; AI-Ishaq et al., 2015), the biofilm phase variation, and biofilm alteration in its behavior and phenotypes, during inter-kingdom relationship, which might promote pro-inflammatory responses (Chia et al., 2008; Afonso et al., 2019). Moreover, staphylococcal PIA may increase inflammatory responses during co-infection with other organisms (Nguyen et al., 2020), as the mixed staphylococcal biofilms with *C. albicans* enhance mortality of *Caenorhabditis elegans*, a worm infection model (Holt et al., 2017). However, SE > CT biofilms did not potently upregulate *TLR-2*, *TLR-4*, and *TLR-6*, suggesting the activation of biofilm molecules through other pathways. More mechanistic studies on biofilm structures are interesting.

**Figure 8:**
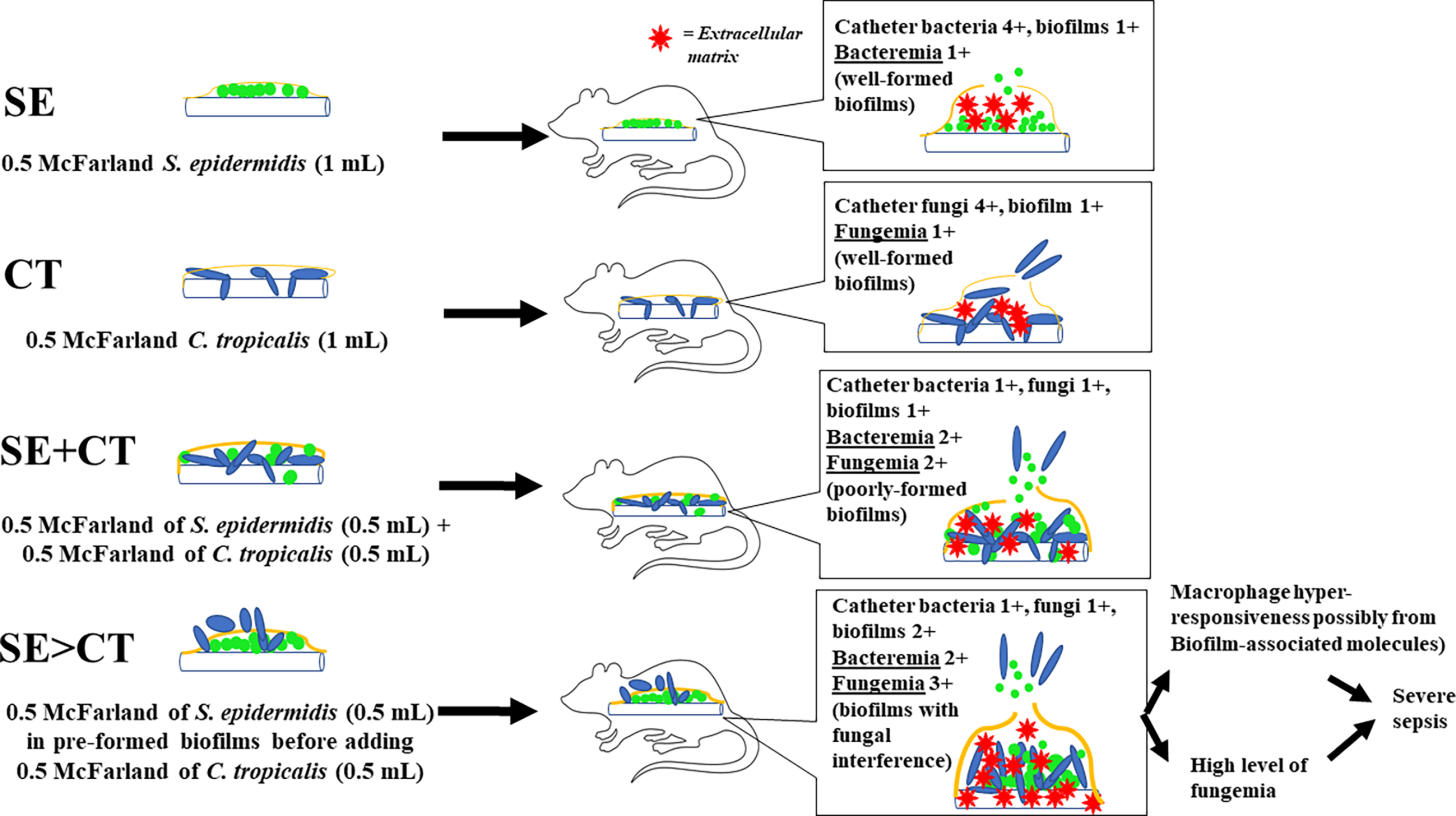
The working hypotheses are i) the well-formed biofilms in catheters with the single organism, *Staphylococcus epidermidis* (SE) or *Candida tropicalis* (CT), consisting of high microbial burdens in catheters (4+) with less biofilm matrix (1+) leading to the low microbial burdens in blood (1+); ii) the poorly formed biofilms in SE simultaneously mixed with CT (SE + CT), consisting of lower organisms in catheters (1+) with similar biofilm matrix (1+), resulting in higher blood microbial burdens (2+) compared with single-organism biofilms but not enough to induce severe sepsis; and iii) fungal interference upon preformed SE biofilms stimulates ECM synthesis of SE with less stable biofilm structure causing higher fungemia (3+), macrophage hyper-responsiveness, and severe sepsis than other groups.

Despite an uncertain mechanism of the potent macrophage responses against SE > CT biofilms, macrophage hyper-responsiveness might partly be associated with sepsis severity. Among all groups, only mice with SE > CT biofilms demonstrated mortality with the highest serum pro-inflammatory cytokines (TNF-α and IL-6), organ injury (kidneys and livers), and fungemia, but not bacteremia. Then, the high fungemia and cytokines in the SE > CT group might be responsible for more severe sepsis, supporting the severity of *C. tropicalis* candidemia in patients (Kang et al., 2017; Sasani et al., 2021). On the other hand, fungemia in SE + CT mice was less in the SE > CT group, which implied the strongest of SE + CT biofilm structures. Perhaps, *Candida* attachment and hyphae interfered with preformed SE biofilms in the SE > CT group, while *Candida* attachment on catheter surface in SE + CT biofilms produced a stronger biofilm structure (**Figure 8**). As such, SE > CT procedure induced thicker, but less stable, biofilms than did other groups, allowing the easy release of toxic biofilm components and fungemia that induced prominent macrophage responses and severe sepsis. Although some organisms in biofilms might be in the non-culturable status (Moser et al., 2021), the culturable organisms in SE > CT and SE + CT biofilms were lower than in the monomicrobial biofilms. Subsequently, the macrophage responses against mixed-organism biofilms (SE + CT and SE > CT) were higher when compared with single-organism biofilms, indicating the possible potent responses toward biofilms structural molecules. Nevertheless, N-acetyl-l-cysteine (l-cysteine), a thiol-containing cysteine with antibiofilms and mild microbicidal activities, attenuated biofilms in all groups of our experiments, supporting previous publications (Olofsson et al., 2003; Dinicola et al., 2014; Phuengmaung et al., 2020). Despite a very high concentration of l-cysteine here, l-cysteine is a non-toxic substance (Spagnuolo et al., 2006), and an accidental leak of l-cysteine into systemic circulation from the catheter lock solutions is harmless in patients (Dinicola et al., 2014). Therefore, the prevention of biofilms by l-cysteine in patients with candidemia and chronic catheterization is clinically interesting. Further studies are warranted.

In conclusion, enhanced biofilm production and biofilm interference by the presence of *C. tropicalis* on preformed *S. epidermidis* biofilms increased candidemia, systemic inflammation, and severe sepsis. In addition, l-cysteine is an interesting agent for prevention of mono- and multi-organism biofilms, which should be further tested.

## Data Availability Statement

The original contributions presented in the study are included in the article/**Supplementary Material**. Further inquiries can be directed to the corresponding authors.

## Ethics Statement

The animal study was reviewed and approved by The Institutional Animal Care and Use Committee of the Faculty of Medicine, Chulalongkorn University, Bangkok, Thailand, following the National Institutes of Health (NIH), USA.

## Author Contributions

PP designed and coordinated all the experiments, performed the *in vitro* and *in vivo* experiments, and wrote and approved the manuscript. WP performed the *in vitro* and *in vivo* experiments and approved the manuscript. US performed the *in vitro* and *in vivo* experiments and approved the manuscript. TC supervised the *in vitro* experiments and approved the manuscript. CC supervised the *in vitro* and *in vivo* experiments and approved the manuscript. AL designed and coordinated all the experiments, analyzed all of these experiments, and wrote the manuscript and approved. All authors contributed to the article and approved the submitted version.

## Funding

This research was supported by Program Management Unit for Human Resources & Institutional Development Research and Innovation-CU [Global Partnership B16F630071 and Flagship B05F630073], TSRI Fund (CU_FRB640001_01_23_1), National Research Council of Thailand (NRCT5-RGJ63001) and Fundamental Fund 2565. PP was supported by Second Century Fund (C2F) for Postdoctoral Fellowship, Chulalongkorn University.

## Conflict of Interest

The authors declare that the research was conducted in the absence of any commercial or financial relationships that could be construed as a potential conflict of interest.

## Publisher’s Note

All claims expressed in this article are solely those of the authors and do not necessarily represent those of their affiliated organizations, or those of the publisher, the editors and the reviewers. Any product that may be evaluated in this article, or claim that may be made by its manufacturer, is not guaranteed or endorsed by the publisher.
