# Supplementary material for: Presence of Candida tropicalis on Staphylococcus epidermidis Biofilms Facilitated Biofilm Production and Candida Dissemination: An Impact of Fungi on Bacterial Biofilms
Source: Front Cell Infect Microbiol. 2021 Oct 22;11:763239. doi: 10.3389/fcimb.2021.763239 (PMC8569676; doi:10.3389/fcimb.2021.763239)
Supplement: Supplementary file 1 [file Table_1.docx]

**Supplementary Table S1** Minimal inhibitory concentration (MIC) and Minimal inhibitory biofilm inhibitory concentration (MBIC) of l-cysteine against several organisms in the study.

| Strain | l-Cysteine (mM) | |
| --- | --- | --- |
|  | **Minimal inhibitory concentration (MIC)** | **Minimal biofilm inhibitory concentration (MBIC)** |
| *S. epidermidis* ATCC12228  *S. epidermidis**  *Candida tropicalis** | 3.125  25  25 | 3.125  50  50 |

***, Clinical isolated strains
