# Supplementary material for: Presence of Candida tropicalis on Staphylococcus epidermidis Biofilms Facilitated Biofilm Production and Candida Dissemination: An Impact of Fungi on Bacterial Biofilms
Source: Front Cell Infect Microbiol. 2021 Oct 22;11:763239. doi: 10.3389/fcimb.2021.763239 (PMC8569676; doi:10.3389/fcimb.2021.763239)
Supplement: Supplementary file 2 [file Table_2.docx]

**Supplementary Table S2** Quantitative real-time PCR primers.

| Target gene | Oligonucleotide sequence (5′−3′) |
| --- | --- |
| Extracellular matrix binding protein (*embp*)  Intercellular adhesion protein A (*icaA*)  Intercellular adhesion protein D (*icaD*)  Intercellular adhesion protein B (*icaB*)  Intercellular adhesion protein C (*icaC*)  Intercellular adhesion protein R (*icaR*)  Transcriptional regulator (*sarA*)  *16S rRNA*  Toll-like receptor 2 (*TLR2*)  Toll-like receptor 4 (*TLR4*)  Toll-like receptor 6 (*TLR6*)  Tumor necrosis factor α (*TNF-α*)  Interleukin-6 (*IL-6*)  Interleukin-10 (*IL-10*)  *β−actin* | **F-**CTACAACAAGCAAGTGCAAC  **R-**TAGAAGTGCTCTAGCATCAT  **F-**ATGCATGTATTTAACTTTTTA  **R-**TTACCGTTGGATATTGCCTC  **F-**ATGGTCAAGCCCAGACAGAG  **R-**TCATATGTCACGACCTTTC  **F-**ATGAAACCTTTCAAATTAA  **R-**TCATCGAATCCGTCCCATTC  **F-**ATGAAGAAAAATAAACTTGA  **R-**TTAATTCCAGTTAGGCTG  **F-**TTGAAAGATAAGATTATTGA  **R-**TACATTTAACAGTGAATATAC  **F-**AATTCTATCCCTTCAAAACC  **R-**TTGTGAATGGTAAGTATGAC  **F-**GATGAACGCTGGCGGCGTGC  **R-**CAATCATTTGTCCCACCTTC  **F-**TCCTCCAATCAGGCTTCTCTGTCTT  **R-**CTCGCAGTTCCAAACATTCC  **F-**CACAGACTTGCGGGTTCTAC  **R-**AGGACCGACACACCAATGATG  **F-**GGCCCTGCCCATCTGTAAGG  **R-**ACTCTCAACCCAAGTGCAGT  **F-**CTCTTCTGCCTGCTGCACTTTG  **R-**ATGGGCTACAGGCTTGTCACTC  **F-**ATGAACTCCTTCTCCACAAGC  **R-**GTTTTCTGCCAGTGCCTCTTTG  **F-**TCTCCGAGATGCCTTCAGCAGA  **R-**TCAGACAAGGCTTGGCAACCCA  **F-**CCTGGCACCCAGCACAAT  **R-**GCCGATCCACACGGAGTACT |
